# Correlation between maturation, growth spurt, and the progression of adolescent idiopathic scoliosis

**DOI:** 10.1186/1748-7161-8-S1-O12

**Published:** 2013-06-03

**Authors:** C Coillard, AB Circo, CH Rivard

**Affiliations:** 1 Ste-Justine Hospital, Montreal, Canada

## Background

The tendency is to start conservative treatment at a lesser Cobb angle, but this can be warranted only if the scoliosis is progressing. Duval-Beaupère [1,2] (1971, 1984) concluded that an “evolutive scoliosis” is progressing in a linear way during two periods: one from birth until the onset of puberty, and the second one during puberty until maturation. Changes in the progression occur suddenly between these two phases (P point) as the first minor signs of puberty occur. Each patient has their own evolution rate; therefore, using clinical signs to determine the exact maturation state of a scoliotic patient and the risk of progression can be difficult.

## Aim

The purpose of this retrospective cohort study was to verify if the scoliosis is indeed progressing in a linear way, and to find a correlation between the progression of the scoliosis, maturation and growth spurt.

## Study design

Out of 1,310 patients treated using the SpineCor orthoses, only the patients that had a Risser sign 0 where considered for this study. 306 patients (out of 806 with Risser 0) finished the treatment and arrived at skeletal maturity. For each patient, we recorded the Height, Risser sign and Cobb angle every 6 months. Correlation between the growth, maturation and the progression of the scoliosis was done using a mix model (linear regression).

## Results

The scoliosis is indeed progressing in a linear way, but we found two main periods of progression: the first period is around Risser 1 (± 6 month), followed by a second risk period just before Risser 4 (United States grading system). The standing height was found to be essentially dependent of the curve progression; for every Cobb augmentation, we found a diminution, or stagnation, for the height (growth). The progression of the scoliosis, and the growth spurt, are directly correlated to the Risser sign.

## Conclusions

The evolution of scoliosis is indeed bound to maturation. Although there are two main periods of progression, in the first period (just before Risser 1) the progression is much more important, and therefore, the evolution with treatment during this period determines the outcome.
